# Cortical Microinfarcts and White Matter Connectivity in Memory Clinic Patients

**DOI:** 10.3389/fneur.2019.00571

**Published:** 2019-06-05

**Authors:** Doeschka Ferro, Rutger Heinen, Bruno de Brito Robalo, Hugo Kuijf, Geert Jan Biessels, Yael Reijmer

**Affiliations:** ^1^Brain Center, University Medical Center Utrecht, Department of Neurology, University Medical Center Utrecht, University Utrecht, Utrecht, Netherlands; ^2^Image Sciences Institute, University Medical Center Utrecht, University Utrecht, Utrecht, Netherlands

**Keywords:** microinfarcts, cerebral small vessel disease, vascular cognitive impairment, white matter connectivity, diffusion tensor imaging

## Abstract

**Background and purpose:** Cerebral microinfarcts (CMIs) are associated with cognitive impairment and dementia. CMIs might affect cognitive performance through disruption of cerebral networks. We investigated in memory clinic patients whether cortical CMIs are clustered in specific brain regions and if presence of cortical CMIs is associated with reduced white matter (WM) connectivity in tracts projecting to these regions.

**Methods:**164 memory clinic patients with vascular brain injury with a mean age of 72 ± 11 years (54% male) were included. All underwent 3 tesla MRI, including a diffusion MRI and cognitive testing. Cortical CMIs were rated according to established criteria and their spatial location was marked. Diffusion imaging-based tractography was used to reconstruct WM connections and voxel based analysis (VBA) to assess integrity of WM directly below the cortex. WM connectivity and integrity were compared between patients with and without cortical CMIs for the whole brain and regions with a high CMI burden.

**Results:**30 patients (18%) had at least 1 cortical CMI [range 1–46]. More than 70% of the cortical CMIs were located in the superior frontal, middle frontal, and pre- and postcentral brain regions (covering 16% of the cortical surface). In these high CMI burden regions, presence of cortical CMIs was not associated with WM connectivity after correction for conventional neuroimaging markers of vascular injury. WM connectivity in the whole brain and WM voxels directly underneath the cortical surface did not differ between patients with and without cortical CMIs.

**Conclusion:**Cortical CMIs displayed a strong local clustering in highly interconnected frontal, pre- and postcentral brain regions. Nevertheless, WM connections projecting to these regions were not disproportionally impaired in patients with compared to patients without cortical CMIs. Alternative mechanisms, such as focal disturbances in cortical structure and functioning, may better explain CMI associated cognitive impairment.

## Introduction

Cerebral microinfarcts (CMIs) are small (<5 mm) ischemic lesions that are increasingly recognized as a clinically relevant marker in stroke and dementia (1). Besides post-mortem detection at autopsy, CMIs can now also be detected *in vivo* on MRI as c*hronic cortical* CMIs on T1-weighted MRI and *acute* CMIs on diffusion-weighted MRI (2).

Both pathology and MRI studies have found a consistent association between CMI presence and cognitive impairment, also after adjustments for the presence of co-occurring Alzheimer's disease (3) and conventional neuroimaging markers of vascular injury (4–7). Although these findings suggest that CMIs play a causative role in the process of cognitive decline, the exact mechanism by which CMIs and cognitive impairment are linked is not yet clear.

Several manifestations of cerebral small vessel disease (SVD), such as white matter hyperintensities (WMHs), lacunes, and cerebral microbleeds have been suggested to affect cognitive functioning by disruption of the WM network (8–12). It appears that the severity and location of these SVD lesions determine their impact on the brain network and consequently cognition (12, 13). Disruption of WM connectivity may also play a role in the relation between cortical CMIs and cognitive impairment. We hypothesized that cortical CMIs exert their effect on the brain network by secondary degeneration of connecting WM pathways. A small study with cerebral amyloid angiopathy (CAA) patients showed that acute subcortical CMIs were indeed associated with changes in the surrounding local WM microstructural integrity (14). Whether similar effects on WM connectivity occur in relation to chronic cortical CMIs is unknown.

We have previously reported that presence of CMIs in memory clinic patients with vascular brain injury is associated with other neuroimaging markers of vascular injury, a diagnosis of vascular dementia and reduced performance in multiple cognitive domains (4). In the present study we investigated whether cortical CMIs in this cohort predominantly occur in specific brain regions and if presence of cortical CMIs is associated with impaired WM connectivity in tracts projecting to these regions.

## Methods

### Study Population

This study involved patients from the TRACE-VCI cohort of the University Medical Center (UMC) Utrecht, an observational prospective cohort study of memory clinic patients with vascular brain injury (i.e., possible VCI) recruited between September 2009 and December 2013 [details described previously (4, 15)]. Patients were included in the cohort if they presented with cognitive complaints at the memory clinic, and had evidence of vascular brain injury on MRI, operationalized as: (1) WMHs with a Fazekas scale grade ≥ 2 (16); (2) ≥ 1 lacunar or non-lacunar infarcts; (3) ≥ 1 cerebral microbleeds; (4) ≥ 1 intracerebral hemorrhage(s) or (5) Fazekas scale grade 1 combined with ≥ 2 vascular risk factors (15). In line with proposed VCI criteria, patients with possible co-existing neurodegenerative disorders (such as Alzheimer's disease) were included in this study cohort, but patients with primary non-vascular or non-neurodegenerative causes of cognitive dysfunction (e.g., brain tumors, depression) were excluded (15). All patients (*n* = 196) underwent a standardized clinical assessment and 3 tesla brain MRI. Patients were included for the present study if they had complete MRI data, including a diffusion weighted scan (*n* = 177), another 13 patients were excluded due to poor quality of the MRI (*n* = 3) or DTI (*n* = 9, including 2 network outliers) and 1 failure to co-register the AAL-template, resulting in a study population of 164.

Ethical approval was provided by the institutional review board of the UMC Utrecht. All procedures were in accordance with the ethical standards of the responsible committee on human experimentation (institutional and national) and with the Helsinki Declaration of 1975, as revised in 2013. Written informed consent was obtained from all participants prior to any research related procedures.

### Clinical Diagnosis of Cognitive Impairment

Educational level was rated according to the 7-point Verhage scale (17). The Clinical Dementia Rating scale (CDR; range: 0–3) was used to assess the severity of cognitive symptoms and functional deficits (18). The mini-mental state examination (MMSE) in Dutch was used as a global measure of cognitive performance (19).

Severity of cognitive impairment was classified at a multidisciplinary consensus meeting. *No objective cognitive impairment* (NOCI) was defined as cognitive complaints, but without objective cognitive impairment on neuropsychological testing. *Mild cognitive impairment* (MCI) was defined as complaints or deterioration from prior functioning and objective impairment in at least one cognitive domain, but with no or mild impairment of activities in daily living. *Dementia* was defined as deficits in two or more cognitive domains at neuropsychological testing and who experienced interference of these deficits in daily living. Further etiological diagnoses of dementia were made based on internationally established diagnostic criteria (without knowledge of CSF biomarkers) into *vascular dementia (VaD)* (20), *Alzheimer's disease (AD)* (21), or other (i.e., dementia such as Lewy body, primary progressive aphasia, cortical basal syndrome, unknown etc (15).

### MRI

All patients were scanned on a 3 tesla MRI scanner (Philips Achieva or Philips Ingenia [Philips Medical Systems, Best, the Netherlands]). The standardized MRI protocol included a 3D T1-weighted sequence (192 slices, voxel size: 1.00 × 1.00 × 1.00 mm^3^, repetition time (TR)/echo time (TE): 7.9/4.5 ms); the following transversal 2D sequences (48 slices, voxel size: 0.96 × 0.96 × 3.00 mm^3^): T2-weighted turbo spin echo (TSE; TR/TE: 3198/140 ms), T2^*^-weighted (TR/TE: 1653/20 ms), and fluid-attenuated inversion recovery (FLAIR; TR/TE/inversion time: 11000/125/2800 ms); and diffusion-weighted imaging [DWI; 48 slices, voxel size: 1.72 × 1.72 × 2.50 mm^3^, TR/TE: 6600/73 ms, 45 gradient directions with a *b*-value of 1,200 s/mm^2^ and one with a *b*-value of 0 s/mm^2^ (3 averages)].

### Neuroimaging Markers

The following neuroimaging markers were rated according to the STRIVE criteria (22) by or under supervision of a neuroradiologist, who was blinded to the clinical condition of the participants: (1) WMHs on the Fazekas scale (16); (2) Lacunes (presence and number); (3) Cerebral microbleeds (presence and number); (4) Medial temporal lobe atrophy (MTA) using the Scheltens scale averaged for both hemispheres (23).

### Brain Volume Measurements

The following semi-automated workflow was used to obtain brain volumes: (1) automated WMH segmentation of 2D FLAIR images using kNN-TTP (24); (2) lesion-filling of 3D T1 images using SLF toolbox (http://atc.udg.edu/nic/slfToolbox/index.html) for Statistical Parametric Mapping 12 (SPM Wellcome Department of Cognitive Neurology, Institute of Neurology, Queen Square London) with default settings (25, 26); (3) default settings were used to obtain probabilistic segmentations for gray matter, WM, and CSF. Total brain volume was defined as the sum of the gray and WM volume. Brain volumes were expressed as a percentage of the total intracranial volume.

### Rating of Cortical CMIs

Cortical CMIs were rated by visual inspection according to previously proposed criteria (2, 27). Cortical CMIs were rated on 3 tesla MRI and were hypointense on T1-weighted imaging, hyper- or isointense on FLAIR or T2-weighted imaging and isointense on T2^*^-weighted imaging. Lesions had to be strictly intracortical and ≤ 4 mm in the greatest dimension on T1. If the lesions measured substantially larger than 4 mm on T2-weighted imaging or within 1 cm proximity of a larger stroke, it was disregarded as the lesion was considered part of a larger stroke. The lesion had to be visible in two viewing planes of the brain (e.g., sagittal, transversal, or coronal plane) and distinct from other structures and lesions such as arteries, veins, enlarged perivascular spaces and cerebral microbleeds. Rating were carried out using MeVisLab (MeVis medical solutions, Bremen, Germany) (28), while the rater was blinded to the clinical condition of the subjects. There was a good intra-rater and interrater (both intra-class correlation coefficient > 0.95) agreement, details regarding the intra- and interrater reliability were published previously (4).

### Cortical CMI Spatial Mapping

**Figure 1:**
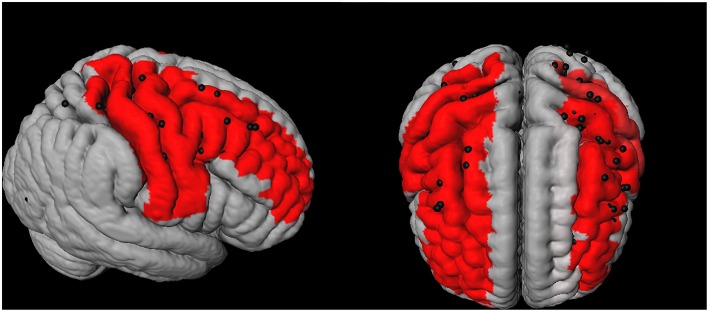
3D representation of the spatial distribution of cortical microinfarcts (CMIs; represented as black dots) across the brain in the cohort. The red areas represent the Automated Anatomical Labeling (AAL)-atlas regions with a high CMI burden (i.e., the 7 brain regions which contained 75% of all the cortical CMIs).

Cortical CMI locations from all patients were registered to Montreal Neurological Institute (MNI) space. The automated anatomic labeling (AAL) template (29) was used as overlay on this sample-averaged CMI map. The number of CMIs within each AAL region was determined to assess whether CMIs predominantly occurred in specific brain regions. The AAL regions with a relatively high number of CMI were defined as *high CMI burden regions*, other AAL regions were defined as *low CMI burden regions*. The threshold for high vs. low CMI burden regions was arbitrarily set at > 5 CMIs (For a histogram of the CMI numbers per AAL region, see Supplementary Figure 1). For 3D rendering of the spatial distribution of cortical CMIs see Figure 1. The volume per AAL region was calculated using automated segmentation using CAT12 after registering the AAL template to the T1 image in patient space.

### Diffusion MRI Processing and Network Reconstruction

**Figure 2:**
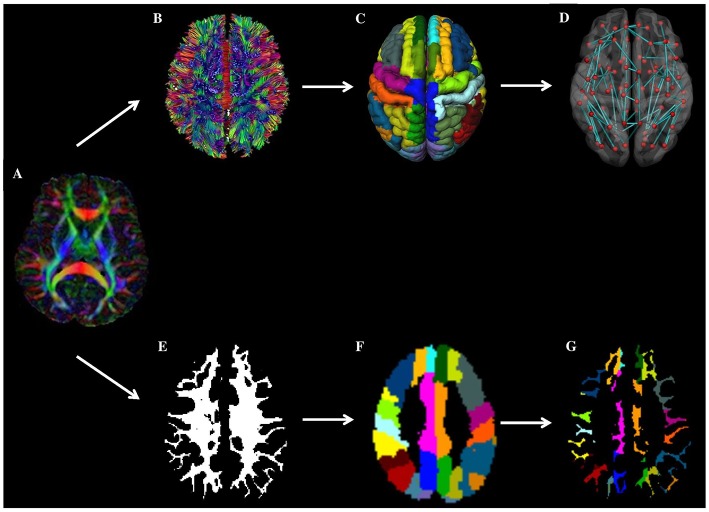
Overview of workflow. In the top panel (network-based approach): from a patients' DTI images (A), WM connections are reconstructed using fiber tractography (B). Next, brain network nodes were defined using the cortical parcellation using the AAL template (C). Subsequently, the structural brain network was reconstructed (D). Weighting of the network was done by multiplying each connection by the mean fractional anisotropy (FA) or mean diffusivity (MD). Finally, the mean FA and MD of connections towards high and low cortical microinfarcts (CMI) burden regions were compared between patients with and without CMIs. In the bottom panel (voxel-based approach), the patient's DTI image A) is combined with the patient's WM segmentation results (E) and AAL template (F) to assess diffusion properties of the WM voxels in the AAL region (i.e. directly underneath the cortex) (G).

Diffusion tensor imaging (DTI) scans were preprocessed as previously described (12, 30) using ExploreDTI version 4.8.6 (www.exploredti.com) and included subject motion correction, unwarping of eddy current and EPI induced distortions and a robust tensor estimation (including adjustment of the B-matrix) (31–33). Next, whole brain deterministic WM tractography was performed using constrained spherical deconvolution (CSD)-based tractography, which is different from standard tensor-based tractography, as it allows reconstruction of crossing fiber pathways (34–36). Reconstruction of fiber tracts was performed by using uniformly distributed starting seed samples throughout the brain's WM at every voxel with a fiber orientation distribution (FOD) > 0.1 (indicating WM) at a 2 × 2 × 2 mm^3^ resolution. Fiber reconstruction was terminated if either a deflection in an angle of more than 45 degrees occurred or if a fiber entered a voxel with a FOD of <0.1 (indicating no WM). An additional terminating mask was not applied. Brain network nodes were defined using the same AAL template as used for the cortical CMI mapping described above, consisting of 90 cortical and subcortical gray matter regions. The AAL template is a commonly used atlas to define nodes in clinical network studies (8, 9, 11). The atlas has the advantage that the gray matter regions also contain a small portion of WM, which allows streamlines that terminate just before the gray-white matter border to be included in the network, thereby reducing the chance of false negative connections. Nodes were considered to be connected if two end points of a reconstructed fiber bundle lay within those nodes, resulting in a 90 × 90 binary connectivity matrix. This matrix was then weighted by multiplying each connection by the mean fractional anisotropy (FA) or mean diffusivity (MD) of that connection, resulting in two weighted-connectivity matrices for each patient. To reduce partial volume effects in WM connections a threshold of FA > 0.2 was applied to all the connectivity matrices. See Figure 2 (upper part A-D) for a graphical representation of this workflow.

### Measures of Whole Brain and Regional WM Connectivity

The Brain Connectivity Toolbox (http://www.brain-connectivity-toolbox.net) was used to calculate network properties, including nodal degree (i.e., number of WM connections per node) and nodal strength (here defined as the mean FA or MD of all WM connections to that node) (37). For this study we used the following constructs: *Whole brain WM connectivity* was assessed by the average FA and MD-weighted nodal strength of all network nodes. *WM connectivity in high and low CMI burden regions* was assessed by the average FA- and MD-weighted nodal strength of the high and low CMI burden regions, respectively, (see paragraph 2.7, for an overview of regions see Figure 1).

### Voxel-Based WM Diffusion Analysis

In addition to the network-based connectivity analyses we also performed a WM voxel-based analysis to assess differences in mean FA and MD. Although we assume that secondary degeneration affects the whole axon running from the cortex to the deep WM, one may speculate that the WM *directly* underneath the CMI containing cortical (i.e., juxtacortical) surface is primarily affected. As can be seen in Figure 2 (lower part) AAL regions mainly consist of GM, but also contain a small WM section in close proximity to the cortical surface. Therefore, we also calculated the mean FA and MD of the WM voxels within each AAL region (using a WM mask with a WM probability threshold of 75). The FA and MD was averaged across all AAL regions for the high and low CMI burden regions respectively, see Figure 2 (lower part E-G) for a graphical representation).

### Statistical Analysis

Differences in baseline characteristics between patients with and without cortical CMIs were analyzed using independent sample *t*-tests (for continuous normally distributed data), χ-square test (for proportions), and Mann-Whitney *U*-test (for continuous, non-normally distributed data). Differences in volume and connectivity strength between brain regions that were identified as high and low CMI burden regions were compared using a paired sample *t*-test (regardless of CMI presence).

The association between the presence of cortical CMIs (predictor) and FA- and MD-weighted WM connectivity (outcome) was analyzed using linear regression and included sex and age (Model 1) and sex, age and conventional neuroimaging markers (WMH Fazekas scale grade 3, presence of lacunar and non-lacunar infarcts) (Model 2) as covariates. Beta values are reported with 95% confidence interval (CI) and corresponding *t*-values and degrees of freedom (df). These analyses were carried out separately for whole brain, high and low CMI burden regions. Within the group of patients with cortical CMIs, patients with 1 vs. patients with multiple cortical CMIs (predictor) were compared on WM connectivity (outcome) using an independent *t*-tests and corresponding df. Using a voxel based approach, the association between cortical CMI presence (predictor) and the mean FA and MD of WM voxels in close proximity to the cortex (outcome) was analyzed using linear regression, adjusted for age and sex. A possible interaction effect between cortical CMI presence and clinical diagnosis on WM connectivity was explored in a regression analysis with *post hoc* Helmert contrasts, where each clinical diagnosis (except the first) was compared to the main effect of all previous diagnoses. *Post-hoc* power analysis was carried out using G^*^Power (Heinrich- Heine-University, Dusseldorf, Germany) (38). All analyses were carried out using IBM SPSS statistics (version 22). A *p*-value of < 0.05 was considered significant, *p-*values were not adjusted for multiple comparisons, as all analyses were planned (not *post-hoc*).

### Data Availability Statement

Any data on the VCI cohort used in these analyses that is not published within this article is available by request from any qualified investigator.

## Results

### Baseline Characteristics of Patients With and Without Cortical CMIs

The 164 patients had a mean age of 72 (± 11) years and 88 (54%) were male. A total of 134 cortical CMIs were detected in 30 (18%) of the 164 patients. The number of cortical CMIs per patient ranged between 1 and 46, 14 patients had 1 cortical CMI and 16 patients had 2 or more cortical CMIs. Baseline characteristics of patients with and without cortical CMIs are presented in Table 1. We have previously published the detailed cognitive profile of patients with cortical CMIs in this specific cohort (4). In short patients with cortical CMIs were more often male, had more non-lacunar infarcts and were more often diagnosed with vascular dementia (all *p* < 0.05).

**Table 1:** Characteristics of patients with and without cortical CMIs.

	**Cortical CMI absent (*N* = 134)**	**Cortical CMI present (*N* = 30)**
**DEMOGRAPHICS**		
Age (years)	72 ± 11	71 ± 11
Sex (males)	67 (50)	21 (70)^*^
Level of education (7 categories)	5 [4-6]	5 [4-6]
**Cognitive Performance**		
MMSE (*n* = 161)	26 ± 3	25 ± 3
CDR	0.5 [0.5–1]	0.5 [0.5–1]
**Clinical diagnosis** (*n* = 154)		
NOCI	24 (19)	3 (11)
MCI	49 (39)	7 (25)
Alzheimer's dementia	48 (38)	13 (46)
Vascular dementia	5 (4)	5 (18)^*^
Other^a^	8 (6)	2 (7)
**NEUROIMAGING MARKERS**		
Total brain volume (% of TIV)	68 ± 4	67 ± 3
Gray matter volume (% of TIV)	36 ± 2	35 ± 2
WMH (Fazekas scale)	2 [1-2]	2 [1-2]
Presence of non-lacunar infarcts	26 (19)	19 (63)^**¥**^
Presence of lacunar infarcts	43 (32)	12 (40)
Presence of cerebral microbleeds	46 (35)	10 (35)

*CMI, Cortical microinfarct; MMSE, mini-mental state examination; CDR, Clinical dementia rating scale; NOCI, No objective cognitive impairment; MCI, Mild cognitive impairment; TIV, total intracranial volume; WMH, White matter hyperintensities*.

a *Other: includes dementia such as Lewy body, primary progressive aphasia, cortical basal syndrome, unknown etc. Data presented as mean ± SD, n (percentages) or median [interquartile range]*.

* *p < 0.05 ^¥^p < 0.0001*.

### Characteristics of High and Low CMI Burden Regions

The spatial location of the cortical CMIs was highly clustered, as more than 70% (*n* = 99) of all cortical CMIs were located within 7 AAL regions (*High CMI burden regions*: middle frontal and pre- and postcentral regions of both hemispheres and the right superior frontal region; Figure 1). The other 83 supratentorial brain regions (i.e., *low CMI burden region)* contained the remaining 37 cortical CMIs. The mean volume of the high CMI burden regions was 68 ± 8.5 ml (16% of total cortical GM volume) compared to 349 ± 41 ml of the low CMI burden regions. Network analyses showed that the high CMI burden regions were more highly connected to the rest of the network than the low CMI burden regions. This was reflected in a higher nodal degree (high burden: 27.2 ± 4.1 vs. low burden: 24.0 ± 2.8), higher FA-weighted nodal strength (high burden:0.300 ± 0.020 vs. low burden:0.293 ± 0.016) and higher MD-weighted nodal strength (high burden:0.940 × 10^−3^ mm^2^/s ± 0.059 vs. low burden:0.985 × 10^−3^ mm^2^/s ± 0.059 all comparisons *p* < 0.0001).

### Association Between Cortical CMI Presence and WM Connectivity

The presence of cortical CMIs was not associated with whole brain FA- and MD-weighted WM connectivity (Table 2). Within the group of patients with cortical CMIs, the number of cortical CMIs (cortical CMI = 1 vs. cortical CMI ≥ 2) also was not related to whole brain FA- (*t*_(df = 28)_ = −0.71, *p* = 0.485) or MD-weighted WM connectivity (*t*_(df = 28)_ = 0.05, *p* = 0.964). Regional analyses showed that in the high CMI burden regions, patients with cortical CMIs had marginally higher MD-weighted WM connectivity (reflecting greater WM disruption), although not statistically significant (*p* = 0.071) while a similar FA-weighted connectivity was observed (Table 2). These association remained non-significant when conventional neuroimaging markers of vascular injury were entered as covariates in the model (Model 2; Table 2). Within the low CMI burden regions, cortical CMI presence was not associated with FA or MD-weighted WM connectivity (Table 2).

**Table 2:** Association between cortical CMI presence and whole brain and regional FA- and MD-weighted WM connectivity in high and low CMI burden regions.

	**Cortical CMI absent (*N* = 134)**	**Cortical CMI present (*N* = 30)**	**Model 1**			**Model 2**		
			**Beta [95% CI]**	***t*-value**	***p***	**Beta [95% CI]**	***t*-value**	***p***
**WHOLE BRAIN**								
FA	0.294 ± 0.017	0.290 ± 0.017	−0.093 [−0.256;0.070]	–1.19	0.234	−0.052 [−0.234;0.104]	−0.69	0.490
MD^a^	0.979 ± 0.057	0.993 ± 0.061	0.087 [−0.047;0.228]	1.27	0.208	0.018 [−0.108;0.138]	0.26	0.795
**HIGH CORTICAL CMI BURDEN REGIONS**								
FA	0.301 ± 0.020	0.296 ± 0.021	−0.109 [−0.254;0.036]	−1.40	0.165	−0.059 [−0.216;0.098]	−0.78	0.440
MD^a^	0.936 ± 0.057	0.958 ± 0.066	0.136 [−0.013;0.285]	1.82	0.071	0.030 [−0.102;0.162]	0.41	0.683
**LOW CORTICAL CMI BURDEN REGIONS**								
FA	0.294 ± 0.016	0.290 ± 0.016	−0.091 [−0.228;0.068]	−1.16	0.247	−0.051 [−0.204;0.102]	−0.67	0.501
MD^a^	0.983 ± 0.058	1.000 ± 0.063	0.082 [−0.050;0.208]	1.20	0.231	0.017 [−0.102;0.130]	0.24	0.808

*CMI, Cerebral microinfarct; FA, Fractional anisotropy-weighted WM connectivity; MD, Mean diffusivity-weighted WM connectivity. Lower FA and higher MD indicated impaired WM connectivity*.

a *MD values × 10^–3^ mm^2^/s*.

*Model 1: Covariates age and sex (degrees of freedom = 160)*.

*Model 2: Covariates sex, age, WMH Fazekas grade 3, presence of lacunar and non-lacunar infarct (degrees of freedom = 157)*.

Since not all cortical CMIs were located in the high burden regions, a sensitivity analysis was performed between patients who had CMIs *exclusively* in the high burden regions (*n* = 20) and patients without CMIs, which yielded similar results.

A *post-hoc* power analysis for CMI presence in high CMI burden regions indicated a power of 0.24 for FA- and 0.44 for MD-weighted connectivity.

### Voxel-Based WM Analysis

Limiting our analysis to WM voxels in close proximity to the cortex showed similar results, i.e., the presence of cortical CMIs was not associated with abnormal mean FA and MD in high CMI burden regions [FA: *t*_(df = 158)_ = −1.01, *p* = 0.314; MD: *t*_(df = 158)_ = 0.753, *p* = 0.452] or in low CMI burden regions [FA: *t*_(df = 158)_ = −0.97, *p* = 0.336, MD: *t*_(df = 158)_ = 1.28 *p* = 0.204].

### Association Between Clinical Diagnosis, WM Connectivity and Cortical CMI Presence

**Figure 3:**
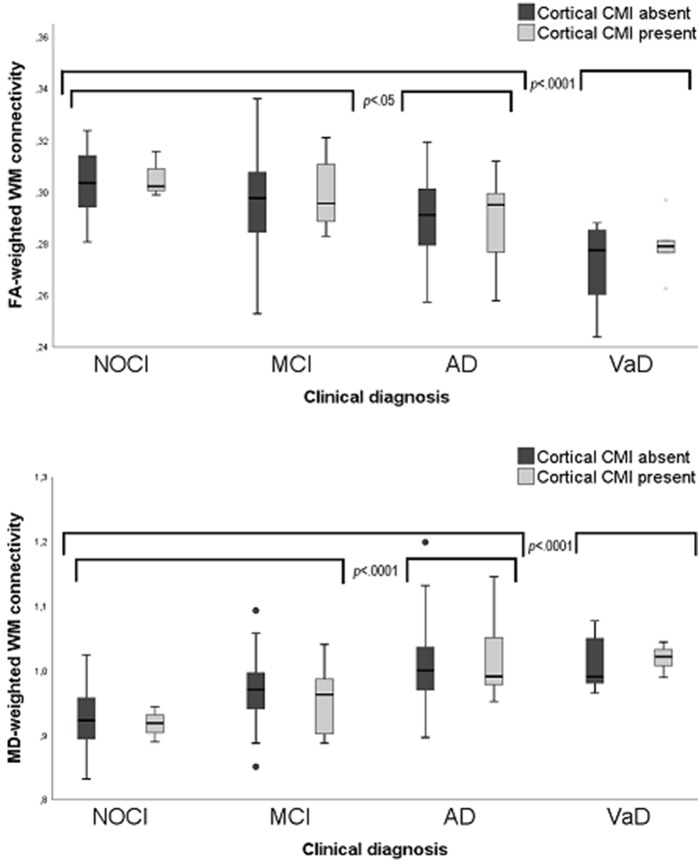
Box plots of FA-(upper) and MD-(lower) weighted WM connectivity between patients with and without cortical CMIs (labels) clinical diagnosis (X-axis). MD values × 10^−3^ mm^2^/s. CMI, Cortical microinfarct; NOCI, No objective cognitive impairment; MCI, Mild cognitive impairment; AD, Alzheimer's disease; VaD, Vascular dementia.

Clinical diagnosis (NOCI, MCI, AD, or VaD) was a significant predictor of whole brain FA- [*F*_(df = 4, 152)_ = 13.9, *p* = 0.005) and MD-weighted WM connectivity [*F*_(df = 4, 152)_ = 10.2, *p* = 0.008]. *Post-hoc* analyses revealed that this effect was driven by the patients with the most severe clinical diagnosis, i.e., patients with AD and VaD had abnormal WM connectivity compared to the other groups (Figure 3). No significant interaction was observed between cortical CMI presence and clinical diagnosis on FA- or MD-weighted WM connectivity [*F*_(df = 4, 152)_ = 0.42, *p* = *0.7*83] or MD [*F*_(dfc = 4, 152)_ = 0.67, *p* = 0.700], indicating that the association between cortical CMI presence and WM connectivity did not differ across the various clinical diagnoses. In a sensitivity analysis of patients without dementia (*n* = 83) presence of cortical CMIs was also not associated with whole brain FA [*t*_(df = 79)_ = 0.43, *p* = 0.667] or MD [*t*_(df = 79)_ = −0.92, *p* = 0.359).

## Discussion

This study shows that cortical CMIs in memory clinic patients vascular brain injury display a strong spatial clustering, as more than 70% of the cortical CMIs were located in frontal, precentral, and postcentral brain regions covering only 16% of the cortical surface. These high CMI burden regions proved to be strongly connected with the rest of the network. However, we found no evidence that the actual presence of cortical CMIs was related to disruption of WM connections to either the high CMI burden regions or within the whole brain.

Cortical CMIs showed a strong predilection for the frontal, precentral, and postcentral brain regions. A similar pattern of CMIs has been found in memory clinic patients (6), but also in patients with ischemic stroke (7, 39), Alzheimer's disease (40) and even in patients with CAA, where vessels are typically affected in the posterior brain regions (41). This preferential lesion location is likely to be of etiological significance. A similar predilection for frontal, pre- and postcentral brain regions was observed in patients with post-stroke cognitive impairment, where a thromboembolic origin has been suggested (42). Future research is encouraged to further explore the relation between lesion location and the pathophysiological origin of cortical CMIs using larger study samples.

We hypothesized that cortical CMIs might affect cognitive performance by disruption of cerebral networks. We have previously reported a relationship between cortical CMIs and reduced cognitive performance on multiple domains in this same cohort (4). In the current study we investigated impaired WM connectivity as possible underlying mechanism. As lesion location could be crucial for its effect on the cerebral network (13), regions with high and low CMI burden were compared. We established no convincing relationship between cortical CMIs and WM connectivity, as the association between cortical CMIs and impaired WM connectivity in high CMI burden regions disappeared after correcting for conventional neuroimaging markers of vascular injury. These findings were in line with our voxel based analysis, showing no local disturbances in the WM directly below the cortical surface of high CMI burden regions. Independent of CMI presence, we did find that patients with dementia, especially VaD, presented with impaired WM connectivity, which corresponds to the known association between network disruption and cognitive deficits (43).

Previous studies in patients with SVD found a disruptive effect of SVD MRI- manifestations, such as WMHs and lacunes, on WM connectivity (8–10, 12, 14, 44–46). Our study is the first to assess the effect of cortical CMIs and did not observe an effect on WM connectivity. This contrasting finding could be explained by the fact that these subcortical manifestations of SVD have a more direct impact on WM integrity, while cortical CMIs are thought to exert their effect indirectly through secondary degeneration. The limited size of the cortical CMIs could also account for the lack of association, as for macroscopic cortical infarcts the size of the lesion is directly correlated to the extent of the axonal injury (47). Considering the average lesion volume of cortical CMIs on 3-T MRI is max 0.1 ml, their effect on WM connectivity could indeed be modest and not of major clinical relevance.

Since cortical CMIs were not related to WM connectivity, other underlying mechanisms should be considered to explain how cortical CMIs affect cognitive impairment. Our earlier work showed that the cortical CMIs were mainly associated with deficits in “cortical” cognitive domains, including visuoconstruction and language (4, 6) suggesting that cortical CMIs potentially affect cognition by disruption of local cortical processes. This notion is supported by a mouse study, that found diminished neural activity and neurovascular coupling in the cortical tissue surrounding the CMI (48). An alternative explanation is that cortical CMIs are a marker of more widespread vascular brain damage that affects cognitive performance (1, 2). As cortical CMIs smaller than 1 mm escape detection on 3 tesla MRI, larger visible cortical CMIs probably only represent the tips of the iceberg. Moreover, it is important to clarify the etiological underpinning of both the detectable as well as these smaller cortical CMIs in order to develop therapeutic strategies that counter cognitive decline.

The strength of our study includes the use of high quality imaging and clinical data of this memory clinic cohort and the systematic approach in cortical CMI rating. Moreover, this study utilized two different DTI approaches to assess the relation with cortical CMIs; a network-based analysis and a voxel-based analysis. However, this study also has some limitations. Firstly, the sample size of cortical CMI cases in our cohort was small, since MRI detectable cortical CMIs occur only in approximately a quarter of memory clinic patients (6). Based on our *post-hoc* power analysis for the observed effect sizes in our study, it would be recommended to replicate results in a larger cohort. Another possible limitation concerns the heterogeneity of the cohort, which includes memory clinic patients with different etiologies, severity of cognitive dysfunction and with large variation in cortical CMI burden. Although this reflects daily clinical practice, it may have reduced our sensitivity to detect abnormalities in WM connectivity due to cortical CMIs.

## Conclusion

We showed that cortical CMIs in memory clinic patients displayed a strong local clustering in frontal and central brain regions, which warrants further investigations into their etiology. Nevertheless, the WM connections projecting to these regions were not impaired in patients with cortical CMIs. This does not support the hypothesis that cortical CMIs affect the brain's integrity through disturbance of WM connections, although further studies, also in larger cohorts with high burden of cortical CMIs, are recommended to confirm our observations.

## Ethics Statement

Ethical approval was provided by the institutional review board of the UMC Utrecht. All procedures were in accordance with the ethical standards of the responsible committee on human experimentation (institutional and national) and with the Helsinki Declaration of 1975, as revised in 2013. Informed consent was obtained from all participants prior to any research related procedures.

## Author Contributions

DF and RH contributed equally to study concept and design, data collection and interpretation, and drafting the manuscript. BdB contributed in data analysis and critical revision of manuscript. HK contributed to image processing and critical revision of manuscript. GB and YR contributed to study concept and design, obtaining funding, interpretation of data, revising the manuscript for important intellectual content.

### Conflict of Interest Statement

The authors declare that the research was conducted in the absence of any commercial or financial relationships that could be construed as a potential conflict of interest.
