# Supplementary material for: Cortical Microinfarcts and White Matter Connectivity in Memory Clinic Patients
Source: Front Neurol. 2019 Jun 5;10:571. doi: 10.3389/fneur.2019.00571 (PMC6560058; doi:10.3389/fneur.2019.00571)

Supplementary Figure 1. Histogram of the number of cortical CMIs per AAL region and allocation of high and low CMI burden regions.

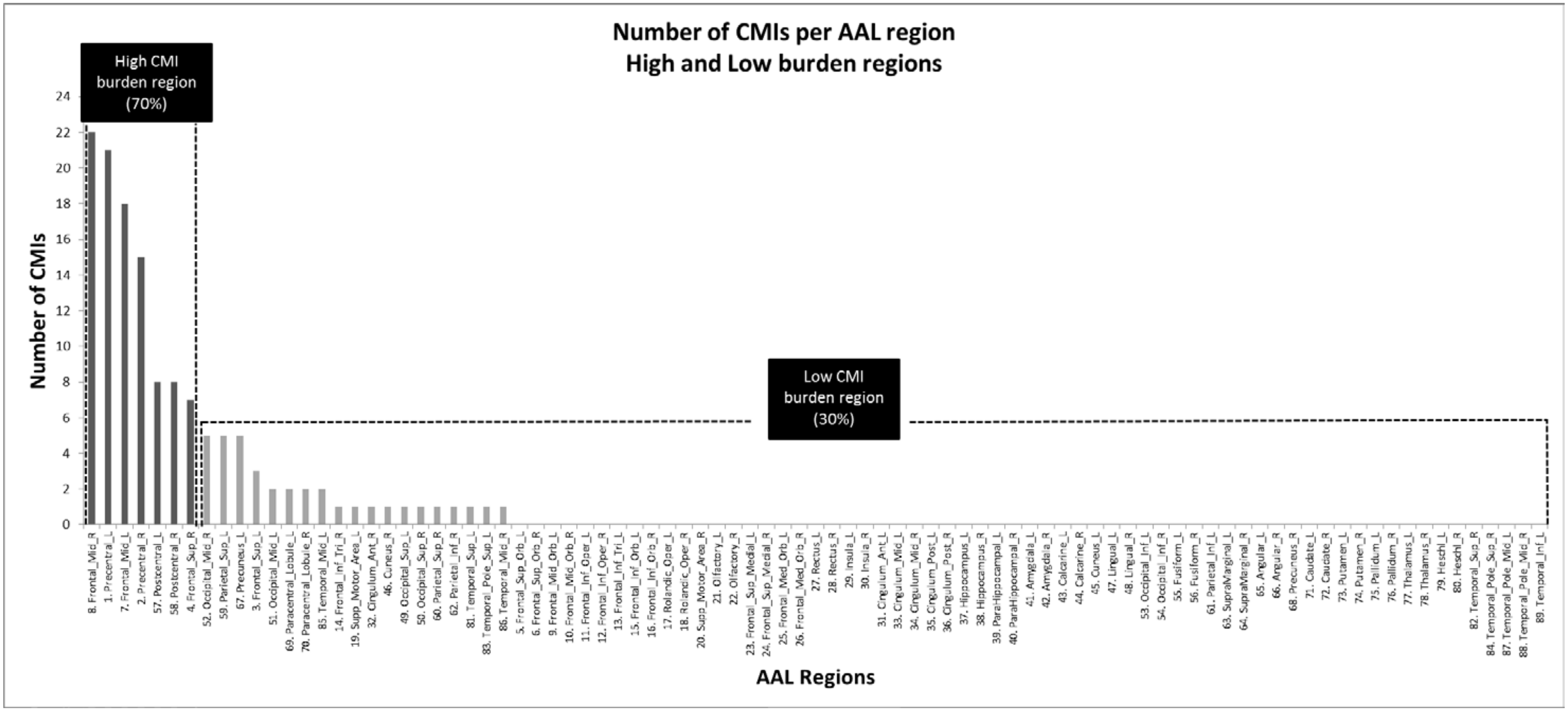

Supplement: Supplementary file 1 [file Data_Sheet_1.PDF]
